# MicroED and cannabinoid research

**DOI:** 10.1186/s42238-021-00070-4

**Published:** 2021-05-11

**Authors:** Crist N. Filer

**Affiliations:** 1 PerkinElmer Health Sciences Inc., 940 Winter Street, Waltham, MA 02451 USA; 2 PerkinElmer, 549 Albany St, Boston, MA 02118 USA

**Keywords:** *Cannabis*, Cannabinoid, MicroED

## Abstract

MicroED has recently emerged as a convenient and powerful tool for the unequivocal structure determination of small molecules and it could likely be used in cannabinoid research as well.

Dear Editor,

Despite the growing application of other spectroscopic tools such as nuclear magnetic resonance (NMR) and Raman spectroscopy, X-ray crystallography is still widely regarded as the definitive gold standard for organic molecule structure determination. However, in the case of cannabinoid research this method has not been fully exploited. A search of the SciFinder® chemistry database produced over 42,000 published articles with the search term “cannabinoid.” However, less than one percent of these publications utilized X-ray crystallography as an analytical tool and most of those few reports employed it to structure elucidate either synthetic cannabinoids (Lu et al. 2005) or *Cannabis* enzyme systems (Shoyama et al. 2005). There have been very few instances where X-ray crystallography was used to characterize a natural product phytocannabinoid of the growing *Cannabis* collection. One example was the structure determination of cannabidiol, first elegantly deciphered by Mechoulam in 1963 with a cogent analysis of NMR chemical shifts and coupling constants (Mechoulam and Shvo 1963). However, it took more than another decade before publication of the conclusive X-ray crystal confirmation of the cannabidiol structure (Jones et al. 1977). With X-ray crystallography often regarded as much an art as science, the explanation for such a delay is perhaps simple. Since many cannabinoids are relatively lower melting solids, the challenge of obtaining appropriately large crystals for conventional X-ray crystallography has been a practical limitation and the likely reason for its lack of routine use. Even in the rare case of higher melting (184−185 **°**C) delta-9-tetrahydrocannabinolic acid B and its X-ray structural determination (Rosenqvist and Ottersen 1975), the authors reported that a suitable prismatic crystal of dimensions 0.45 × 0.30 × 0.12 mm was required and obtained, but only after “slow evaporation of a chloroform solution.”

However, it is possible that with the advent of a very new technical adaptation, crystallography may soon become a more routine analytical method for cannabinoid characterization. First, some background information is useful. In 2013 using an electron cryo-microscope with a diminished electron beam (to avoid crystal damage), Gonen and Nannenga merged it with an improved data collection system and termed this hybrid technology “microcrystal electron diffraction” or MicroED (Nannenga 2020). With MicroED, no longer were arduously obtained large crystals necessary for analysis. Even seemingly amorphous powders often contained enough microcrystals of a substance to support this new method. Since its discovery, MicroEd has become an exceptionally useful tool for the structural analysis of biomolecules like proteins (Nannenga and Gonen 2019). However, importantly for cannabinoid research, a technical breakthrough has recently occurred for MicroED. The application of this powerful method has now been extended to small molecules as well (Jones et al. 2018**;** Nguyen and Gonen 2020). Diverse structures such as acetaminophen, biotin, brucine, carbamazepine, cinchonine, ibuprofen, progesterone, and even heterogeneous mixtures of these have been structurally analyzed to angstrom resolution with this new method.

MicroED would seem to be an entirely new paradigm and transformational technique for the rapid and unambiguous structure determination of small molecules. A literature search revealed that MicroED has not yet been applied to cannabinoid research but it could likely be. However, practitioners have noted that despite its impressive results to date, “the MicroED method has largely gone unnoticed in the small molecule communities” (Jones et al. 2018). It is the purpose of this note to heighten awareness of the potential application of MicroED in cannabinoid research.

## Data Availability

Not applicable**.**

## Abbreviations

MicroED: Microcrystal electron diffraction
NMR: Nuclear magnetic resonance
